# “Platelet-associated regulatory system (PARS)” with particular reference to female reproduction

**DOI:** 10.1186/1757-2215-7-55

**Published:** 2014-05-16

**Authors:** József Bódis, Szilárd Papp, István Vermes, Endre Sulyok, Péter Tamás, Bálint Farkas, Katalin Zámbó, Ioannis Hatzipetros, Gábor L Kovács

**Affiliations:** 1 Department of Obstetrics and Gynecology, University of Pécs, 7624 Pécs Édesanyák útja 17, Hungary; 2 HAS-UP Human reproduction scientific research group, 7624 Pécs Édesanyák útja 17, Hungary; 3 Institiute of Diagnostics, Faculty of Health Sciences, University of Pécs, 7400 Kaposvár, Szent Imre u. 14/b, Hungary; 4 Faculty of Health Sciences, University of Pécs, 7621 Pécs Vörösmarty u. 4, Hungary; 5 Department of Nuclear Medicine, University of Pécs, 7624 Pécs Ifjúság u. 13, Hungary; 6 Department of Laboratory Medicine, University of Pécs, 7624 Pécs Ifjúság u. 13, Hungary; 7 Szentagothai Research Centre, University of Pécs, 7624 Pécs, Ifjúsag u. 20., Hungary

**Keywords:** Platelets, Cardiovascular system, Tumorgenesis, Endocrine organs, Hypothalamo-pituitary-ovarian system

## Abstract

**Background:**

Blood platelets play an essential role in hemostasis, thrombosis and coagulation of blood. Beyond these classic functions their involvement in inflammatory, neoplastic and immune processes was also investigated. It is well known, that platelets have an armament of soluble molecules, factors, mediators, chemokines, cytokines and neurotransmitters in their granules, and have multiple adhesion molecules and receptors on their surface.

**Methods:**

Selected relevant literature and own views and experiences as clinical observations have been used.

**Results:**

Considering that platelets are indispensable in numerous homeostatic endocrine functions, it is reasonable to suppose that a ***platelet-associated regulatory system (PARS)*** may exist; internal or external triggers and/or stimuli may complement and connect regulatory pathways aimed towards target tissues and/or cells. The signal (PAF, or other tissue/cell specific factors) comes from the stimulated (by the e.g., hypophyseal hormones, bacteria, external factors, etc.) organs or cells, and activates platelets. Platelet activation means their aggregation, sludge formation, furthermore the release of the for-mentioned biologically very powerful factors, which can locally amplify and deepen the tissue specific cell reactions. If this process is impaired or inhibited for any reason, the specifically stimulated organ shows hypofunction. When PARS is upregulated, organ hyperfunction may occur that culminate in severe diseases.

**Conclusion:**

Based on clinical and experimental evidences we propose that platelets modulate the function of hypothalamo-hypophyseal-ovarian system. Specifically, hypothalamic GnRH releases FSH from the anterior pituitary, which induces and stimulates follicular and oocyte maturation and steroid hormone secretion in the ovary. At the same time follicular cells enhance PAF production. Through these pathways activated platelets are accumulated in the follicular vessels surrounding the follicle and due to its released soluble molecules (factors, mediators, chemokines, cytokines, neurotransmitters) locally increase oocyte maturation and hormone secretion. Therefore we suggest that platelets are not only a small participant but may be the conductor or active mediator of this complex regulatory system which has several unrevealed mechanisms. In other words platelets are corpuscular messengers, or are more than a member of the family providing hemostasis.

## Introduction

Platelets have a volume of 7.1 ± 4.9 μm^3^, a diameter of 3.6 ± 0.7 μm. They have a surface-connected canalicular system [1,2] and four distinct populations of granules: α-granules (containing β-thromboglobulin, platelet factor 4 (PF4), vWF (von Willebrand factor), thrombospondin, fibrinogen, albumin, IgG, fibronectin, PDGF (platelet-derived growth factor), and factor V [3-5]), dense bodies (containig adenine nucleotides, calcium and magnesium, and serotonin [6,7]), lysosomes, and microperoxisomes. Granules merge with channels of the canalicular system after platelet stimulation and evacuate their contents [8-10]. Platelet have many important surface receptors (among others GP Ib-IX, GPIIb/IIIa, ICAM-2, P-selectin, F11R or JAM-A, TLRs, and chemokine receptors [11,12]). The platelet-activating factor (PAF) is also an important secretory product of platelets mediating the platelet aggregation, inflammation and anaphylaxis [13] (Table 1).

**Table 1:** Platelet surface and soluble molecules

**Surface molecules**	**Soluble molecules**	**Molecules with effect to platelets**
GP Ib-IX	Plasminogen activator inhibitor-1	Fibrinogen
GP IIb-IIIa	α2-antiplasmin	vWF
ICAM-2	Fibrinogen	CD40 ligand
P-selectin	Factor V	Monoclonal antibody F11
F11 receptor	β-thromboglobulins	Leukocyte β2 integrin LFA-1
TLRs	Chemokine RANTES	Leukocyte β2 integrin Mac-1
Chemokine receptors	IL-1α	DC-SIGN
JAM-A	IL-1β	PSGL-1
JAM-C	IL-1RA	hCG
CD63	PF4	Progesterone
Benzodiazepine-receptor	PAF	Estradiol
	EGF	
	PDGF	
	Insulin-like growth factor 1	
	TGFβ	
	Thrombospondin	
	Fibronectin	
	vWF	
	ADP	
	ATP	
	Histamine	
	Serotonin	

GP Ib-IX, Glycoprotein Ib-IX; GP IIb-IIIa, Glycoprotein IIb-IIIa; ICAM-2, Intercellular adhesion molecule 2; TLRs, Toll-like receptors; PF4, Platelet factor 4; PAF, Platelet-activating factor; EGF, Epidermal growth factor; PDGF, Platelet-derived growth factor; TGFβ, Transforming growth factor beta; vWF, von Willebrand Factor; ADP, Adenosine diphosphate; ATP, Adenosine triphosphate; DC-SIGN, Dendritic Cell-Specific Intercellular adhesion molecule-3-Grabbing Non-integrin; PSGL-1, P-selectin glycoprotein ligand-1.

Blood platelets play an essential role in hemostasis, thrombosis and coagulation of blood. The function of platelets in the maintenance of hemostasis has long been recognized and well defined. Beyond these classic functions their involvement in inflammatory, neoplastic and immune processes were also investigated by several reports. Nevertheless, their distinctive armament of soluble mediators as well as the presence of surface receptors suggests that platelets must have further roles in several physiological and pathophysiological regulatory processes [14].

Platelets have specialized adhesion mechanisms that make them able to take part in cell-matrix and cell-cell interactions. This property let platelets arrest bleeding and promote vessel repair. High shear stress in stenotic atherosclerotic arteries cause platelet adhesion leading to the development of arterial thrombosis and cardiovascular events [15]. The connection between platelets and cardiovascular diseases has also been proved by the role of aspirin (by permanent inactivation of COX-1 and −2) in the prevention of cerebrovascular and cardiovascular diseases [16].

It has become clear that platelets are not simply cell fragments that plug the leak in a damaged blood vessel; they are, in fact, also key components in the innate immune system. They express many immunomodulatory molecules and cytokines with the ability to modulate the immune system through interactions with various cells [17]. Uncontrolled or misdirected activation of platelets and the immune response is involved in the onset and progress of several conditions with an inflammatory component, such as coronary artery disease and autoimmune diseases. Direct interaction between platelets and bacteria leading to platelet activation has also been reported in several studies in vitro and in vivo [18,19].

The involvement of platelets and coagulation factors in tumor progression and metastasis has been extensively documented. Tumor cells are known to interact with all major components of the hemostatic system, including platelets. It has been proved that interrelationship between tumor cells and platelets, within the bloodstream, is important during the early phase of metastasis [20]. Cancerous patients have increased risk of thrombosis, and the signs of it are most severe if the disease has progressed to a metastatic stage [21].

The role of platelets in different endocrine diseases have already been published. Hyperthyroidism is associated with increased platelet production [22]. Not only thyroid but also parathyroid disorders are associated with hemostatic abnormalities and vascular diseases [23]. The relationship between platelet function and an increased occurrence of vascular diseases in diabetic patients has also been proven. Hyperglycemia can lead to increased platelet reactivity and not only hyperglycemia but insulin can also directly control platelet function by a functional insulin receptor found on human platelets [24].

## Role of platelets in female reproduction

Platelet aggregation and adhesion to endothelial cells has been observed in the capillaries around periovulatory follicles [25]. Concentrations of TXB2 in the follicular wall and in follicular fluid were dramatically elevated in preovulatory follicles. Aggregates of platelets (the suspected source of TXA2) were adhered to endothelial cells at this time; in some cases intra and extravascular clotting was apparent. It is suggested that platelets and (or) TXA2 might contribute to this periovulatory processes.

The platelet count in the inferior vena cava was significantly decreased 48 h after PMSG administration and was further decreased after hCG administration in immature rats. When both ovaries were extirpated, the administration of PMSG and hCG did not decrease the platelet count. Subcutaneous administration of a PAF antagonist after PMSG injection decreased the number of ova shed in a dose-dependent manner. The decrease in the platelet count induced by the administration of PMSG and hCG was reversed to the level of the untreated control group by a PAF antagonist. This inhibitory activity of a PAF antagonist on ovulation and thrombocytopenia was completely reversed by the injection of a synthetic PAF [26].

Oestrogen has been reported to regulate the plasma level of PAF-acetylhydrolase activity, and its suppression by oestrogen leads to the accumulation of PAF in the ovary [27,28]. It means that PAF synthesis may be mediated by ovarian steroid hormones during ovulation. It is reported that serotonin and histamine have a direct stimulatory effect on ovarian steroid synthesis, and it was suggested that these neurotransmitters may reach the ovary by platelets which assume a platelet-associated paracrine-endocrine regulation [29,30].

There are experimental data demonstrating the presence and actions of various neurotransmitters in the ovary, thus supporting the view that they might play a role in intraovarian regulatory mechanisms, although their exact function in the regulation of ovarian hormone secretion is unclear. We described that serotonin stimulated a dose-related increase in progesterone secretion, however estradiol secretion in response to serotonin was dose-independent. In cell culture, co-administration of serotonin with gonadotropins had no additive effect on gonadotropin-induced secretion of progesterone, while it further augmented that of estradiol. Histamine stimulated a dose-related increase in estradiol secretion with a maximal stimulatory effect. Progesterone production in response to histamine stimulation was independent of dose at the limit of significance, but histamine had no effect on progesterone release. We suggest that histamine has a direct stimulatory effect on steroid production of granulosa cells mediated via the H1-receptor [31,32]. Both serotonin and histamine are originated from platelets and may have a physiological role in the corpus hemorrhagicum, when luteinization is initiated.

The relationship between ovarian function and platelets is also being investigated. The human corpus luteum is a unique endocrine organ that fills the postovulatory follicle during the menstrual cycle. Its role is to provide progesterone to the systemic circulation. Fujiwara used immunohistochemical procedures to show that considerable numbers of platelets and red cells were present at extravascular sites among luteinizing granulosa cells after ovulation. Increased vascular permeability beneath the follicular basement membrane may permit extravasation of blood cells into the spaces around luteinizing cells. The platelets, activated through adhesion to components of the extracellular matrix (collagen?), probably by the same mechanisms involved in hemostasis, expressed P-selectin demonstrating that platelet secretion had occurred. In vitro studies showed that progesterone production by luteinizing granulosa cells from patients undergoing in vitro fertilization therapy was promoted by direct contact with platelets during 4-d culture. In contrast, platelet-derived soluble factors induced both granulosa cell spreading and endothelial cell migration, evidence for multiple actions of platelets in the process of luteinization. An in vivo consequence is enhanced extracellular matrix formation around the luteal cells and the growth of vascular networks. It has been previously shown that luteinizing granulosa cells secrete proangiogenic factors such as vascular endothelial growth factor (VEGF); although VEGF induces endothelial cell proliferation, it does not induce their migration. Interestingly, it has been shown that when cocultured with platelets, granulosa cells inhibited endothelial cell migration. Although this could be interpreted as being contradictory to their hypothesis, the authors suggest a crucial regulatory role of granulosa cells in protecting against “overvascularization” and an excessive stimulatory effect of platelets. The role of platelets in these events was compared by the authors to their role and that of coagulation proteins in inflammation.

It is known that an increase in platelet number in ovarian hyperstimulation syndrome (OHSS) coupled with elevated blood coagulation factors and hyperviscosity in a severe form of this syndrome may result in the development of intravascular thrombosis [33]. OHSS is a severe, potentially lethal iatrogenic disease, resulting from vasoactive products released by hyperstimulated ovaries. OHSS is characterized by increased capillary permeability triggerred by VEGF, which correlates with increased platelet activation [34]. Activated platelets release histamine, serotonin, platelet-derived growth factor, or lysophosphatidic acid. These substances might further drive the pathophysiological cascade leading to OHSS. Based on this we hypothesized that using aspirin might prevent this syndrome [35]. To prove this, we randomly administered aspirin to high risk and low risk patients undergoing in vitro fertilization protocol and determined the proportion of OHSS in each group. In our study, among the patients treated with aspirin, there were only 2 occasions of severe or critical OHSS (0.25%). Both patients belonged to the high risk group. However, among those high risk patients that received no prophylactic aspirin regimen, 43 patients (8.4%) presented OHSS. No OHSS was encountered in the low risk groups. Since there was a significant difference in the occurrence of severe OHSS between the aspirin treated and non-treated high risk groups, an overall advantageous effect of aspirin preventing OHSS might be seen. We strongly advise, therefore, the use of low-dose aspirin to prevent OHSS or to reduce its severity in all cases associated with high risk of that severe complication of ovulation induction.

The coordinated interplay between platelets and ovaries may be accomplished further by the brain-derived neurotrophic factor (BDNF) - Sigma-1 receptor – Akt – eNOS system. Down-regulation of the system has been proved to be a pathogenetic factor for depression, cardiovascular diseases, diabetes mellitus type 2 and for several components of metabolic syndrome. The activation of Sígma-1R signaling has emerged as a link between these pathologies [36,37].

BDNF is mainly produced by the brain accounting for over 70% in the circulation. Additional BDNF sources are the vascular endothelium, smooth muscle cells and activated monocytes/lymphocytes. It readily crosses the blood–brain barrier, circulates in the plasma and is taken up and stored in the platelets. Upon platelet activation/clotting BDNF is released to the extracellular fluid and platelet derived serum BDNF closely correlates with that in the brain [38,39]. Importantly, positive correlation has been documented between BDNF and serotonin release [40,41]. BDNF and serotonin are assumed to act synergistically as serotonin secretion, turnover and signaling is regulated by BDNF, while serotonin in turn controls BDNF production [41].

BDNF and its receptors are widely distributed in peripheral tissues and implicated in modulating ovarium function and diseases related to ovarium dysfunction. With this contention in line Sigma-1R stimulation with selective serotonin reuptake inhibitors (SSRI) in ovariectomized animals and in postmenopausal women improved depressive disorders and provided cardiovascular protection [42]. This beneficial effect of SSRI is achieved by increased conversion of the precursor proBDNF to mature BDNF and its enhanced secretion into the extracellular space [43]. Stimulation of Sigma-1R by ovarial steroids, dehydroepiandrosterone (DHEA) or pregnelolone (PREG) and/or by their sulfates (DHEAS, PREGS) resulted in similar protection in experimental or clinical settings [44].

Interestingly, there are marked cyclic variations in platelet BDNF levels during menstruation. In the second half of menstrual cycle endometrial cells produce substantial amount of BDNF which is taken up by the platelets. The reduced accumulation of endometrium-derived BDNF in the platelets may lead to clinical consequences as seen in menopausa [45].

In a most recent comprehensive review detailed analysis is presented on the importance of BDNF in the paracrine regulation of ovarian function [46]. BDNF has been shown to stimulate the early and final stage of folliculogenesis, oocyte maturation and early embryonic development. In women undergoing IVF treatment it was present in the follicular fluid, and cumulus and granulosa cells were identified as its major source. Significant positive correlation was found between circulating estradiol and BDNF levels and its ovarian secretion could be enhanced with LH, FSH and hCG administration. It is to be noted that BDNF release by the platelets was not mentioned in this particular review, however accumulating evidences suggest that platelet-derived mediators have a prominent role in the intra-ovarian regulation.

The involvement of platelets in the reproductive processes is further supported by the observations that human endometrial tissues and stromal cells in primary culture contain PDGFs and their receptors. They have been shown to induce growth, proliferation, motility and contractility of endometrial stromal cells. It is of note that several factors, including estrogens increase the tissue expression of PDGF-Rs, consequently they enhance the local effects of PDGFs [47].

In the followings we give a short summary of our concept concerning the involvement of platelets in modulating the function of the hypothalamo-hypophyseal-ovarian system. Considering the data, hypothalamic GnRH releases FSH from the anterior pituitary, which induces and stimulates follicular and oocyte maturation and steroid hormone secretion in the ovary. At the same time follicular cells enhance PAF production. Through these pathways activated platelets are accumulated in the follicular vessels surrounding the follicle and due to its released soluble molecules (factors, mediators, chemokines, cytokines, neurotransmitters) locally increases oocyte maturation and hormone secretion.

## Platelets in pregnancy

Platelet’s functions and their role in the coagulation process in pregnancy are well documented, and there are several data to suggest their importance in embryo implantation, placental development, preeclampsia, HELLP syndrome, and other physiological and pathophysiological processes.

### Platelets may play a role in placentation

Circulating platelets stores several bioactive mediators within their granulas, including PDGF-B, bFGF, HGF, IGF, IGF-1, PF4, TSP1, TGF-β1 and VEGF, and they release them upon stimulation. Most of these molecules are found to be relevant in mouse placentation, for instance bFGF, IGF-1 or PDGF which were proved to be an effective protector of trophoblast cells against TNF-α and INF-γ induced apoptosis *in vitro*[48]. Beyond that, the deficit of PDGF-B or PDGF-R-β, results vessel dilation in the placenta [49]. Moreover the developmental failure of the placenta is associated with the absence of lipid-phosphatase 3 (LLP-3) [50]. Such findings implemented the necessity of *in vivo* animal experiments to further investigate the role of platelets in implantation and development. The mouse model of quantitative platelet disorder (NF-E2 deficient mice) and qualitative thrombocyte defect (Gαq, Par-3, or Par-4 deficient mice) makes them eligible for such task.

The NF-E2 transcriptional factor is a crucial protein of the megakaryopoiesis, therefore NF-E2 “knock-out” mice develops a severe quantitative platelet deficiency [51]. Such condition in these animals is associated with significant intrauterine growth retardation (IUGR), explained by the vascular abnormality of the placental labyrinthine layer, due to impaired blood vessel maturity. Interestingly, failure of the placental vascularization is only reported in NF-E2 null embryos, but not in NF-E2 null mothers, nor mice with other platelet disorders. NF-E2 “knock-out” mothers delivers same number of litter, and the embryonic loss was found to be not significantly different compared to wild type mice, despite that histological evaluation revealed numerous spacious blood pools in the placenta (intraplacental hemorrhage) [52,53].

On the other hand, in case of qualitative thrombocyte abnormalities placental disorders can not be revealed. For example Gαq-deficient mice (lack of the α-subunit of the guanine nucleotide binding protein Gq), has normal number of thrombocytes, but their platelets can not be activated, develop normal placental development and embryos [54]. Likewise, when protease-activated receptor 3 and 4 (Par-3 and Par-4) mediated platelet activation is also missing in mice an intact, normal placenta and embryo develops [55].

Next to the experimental results from mice, human data also show that maternal platelets might play important role in the placental vascular remodeling, which assures proper placental perfusion and maintains the pregnancy itself. Such phenomenon is based on the endovascular invasion of trophoblast cells into the maternal spiral arteries, enabling them to transform large-caliber vessels [56]. The mechanism of such alteration is, that maternal platelets are trapped in the lumen of the spiral arteries by endovascular trophoblast aggregates. These activated platelets are communicating with neutrophil granulocytes, by expressing P-selectin on their surfaces. These cell surface cell adhesion molecules are binding to their ligands (P-selectin glycoprotein ligand-1; PSGL-1) on the activated granucolcytes, and in turn they produce PAF to facilitate the further aggregation processes [57]. Extravillous trophoblast cells express chemokine receptor (CCR1), which plays as a functional receptor for trophoblasts. Their ligands (CCR1 ligand) are secreted by the activated platelets in the placenta to encourage the remodeling processes, and to attract more endovascular trophoblasts to the site [58]. To achieve such vascular caliber changes during the early placentation a direct contact between the platelets and the trophoblast cells is not fully required. Therefore it is suspected that some soluble platelet-derived factor(s) are regulating the placental vascular remodeling processes in human [58].

Clinical observations, in case of recurrent pregnancy loss, intrauterine growth restriction and preeclampsia, highlighted the effectiveness of low-dose platelet cyclooxygenase inhibitor (Aspirin®), which reduces the platelet aggregation by suppressing the TXA2 synthesis [59]. However, pregnant women with Bernard-Soulier syndrome or other congenital thrombocytopenias usually have an uncomplicated pregnancy and they deliver a healthy newborn, but the risk for a potential postpartum bleeding is increased [60].

### Platelets in preeclampsia and HELLP syndrome

Pregnancy is a condition of immunological balance between the mother and her semi-allograft fetus. Besides, pregnancy is considered as a controlled pro-coagulation, and a mild inflammatory state. As platelets are involved in all of these processes, their consumption is enhanced. Consequently, platelet production is augmented, resulting in a slightly increased ratio of young platelets in the bloodstream. However, platelet count remains normal (150,000 to 450,000 platelets per microliter) throughout normal pregnancy. This slim equilibrium will be disturbed in conditions where complex maternal *adaptation* to her pregnancy is inadequate.

Preeclampsia is a serious disease in pregnancy with heterogeneous etiology [61,62]. Early-onset, or hypovolemic, or serious preeclampsia and the hemolysis, elevated liver enzymes, and low platelet count (HELLP) syndrome are thought to be conditions due to imbalance between the mother and fetus [63]. Theoretically, the progenitors could be of normal fetal antigens but their pathologically increased mass in the maternal circulation and/or the abnormal maternal reaction to them (“*maladaptation*”) could lead to the clinical symptoms. Platelets play a pivotal role in the holistic defending mechanism, being stimulated in increasing degrees in these serious diseases. Overstimulation collapses this platelet-associated regulation contributing to the development of end-organ damages.

Early-onset preeclampsia can be considered as a 2-stage disease. Abnormal placentation (first stage) through endothelial damage is responsible for the potential of end-organ manifestations (second stage). The first stage is characterized by effects of anti-angiogenic substances such as the soluble fms-like tyrosine kinas-1 (sFlt-1) [64], endoglin (sEng) [65], and human interferon-inducible protein 10 (IP-10 or CXC10) [66] which bind and neutralize different growth factors, placental growth factor (PlGF), VEGF, and TGF-β required for placental and fetal angiogenesis. Agents directly from this shallowly implanted placenta and also from activated leukocytes or platelets may represent the link between abnormal placentation and endothelial inflammatory injury. Recent candidates are the anti-angiogenic agents; free oxygen radicals and some cytokines, first of all TNF-α [67,68], thrombogenic content of miroparticles released from the surface of different cells [69]; syncytiotrophoblast microvilli or vesicles [70] releasing sFlt-1 and sEng; fetal cells and cell-free fetal DNA [71] circulating in a relatively huge amount in preeclamptic maternal bloodstream. Consequently, markers of endothelial injury e. g. fibronectin [72] soluble thrombomodulin [73], or von Willebrand factor [74] show elevated levels in preeclampsia. Similarly, levels of platelet-aggregation products (e.g. β thromboglobulin, PF4, TXA2) are elevated [75].

Epidemiological studies suggested that “immune-maladaptation” may have an etiologic role in the development of preeclampsia [76]. Long (>4 months) maternal ejaculate-exposition results in slow alloimmunization, and development of immuntolerance against the given gentile antibodies [71]. In the absence of appropriate partner-specific mucosal immuntolerance the incidence and severity of preeclampsia will increase [77]. Fetal proteins contact with maternal immune system not only at the feto-maternal interface but also in the maternal circulation, where fetal cells, cell debris, or DNA can be detected in huge number in preeclamptic individuals, showing correlation with the severity of the disease [71]. Platelets play a pivotal role in the initiation and modulation of immunological processes. Soluble platelet-released immune modulators (chemokines, cytokines) coordinate leukocyte migration, endothelial adhesion, and function [78]. There is no doubt that signals of activated platelets are essentials in the development of maternal immune-respose in preeclampsia too.

It is well known for some decades that platelets and also intravascular coagulation cascade are activated in preeclampsia. Damaged endothelial cells produce more adhesive substances (e.g. fibronectin, vascular cell adhesion molecule-1, E-selectin) and possess less antithrombotic capacity (e.g. weak thrombomodulin effects) than normal endothelial cells [73,79]. Impairment of endothelium exposes the subendothelial collagen, a potent platelet-activating agent, to vessel content. Increased nitric oxide (NO) production during normal pregnancy effectively decreases the sensitivity of platelets to pro-aggregating agents [80]. Asymmetric dimethyl arginine (ADMA), the endogenous inhibitor of NO synthesis, levels are increased in preeclampsia [81] lowering the effectiveness of an important protective mechanism of platelet activation. In accordance, the augmentation of endogenous and reactive NO production shows beneficial effects in gestational hypertension [82]. Similarly, damaged endothelial cells produce less prostaglandin I_2_ (PGI_2_ or prostacyclin), another potent agent against platelet activation [83].

Activated platelets not only release the vasoconstrictor TXA_2_, but also increase the expression of adhesive agents [84]. Activated platelets this way enhance further platelet adhesion, and also the endothelium − leukocyte contact and trigger leukocyte arrest and transendothelial migration [85].

Erythrocyte deformability has been found to be decreased in preeclampsia which may contribute to decreased capillary circulation [86], since capillary diameter is about the half of those of red blood cells. Peripheral mechanical hemolysis is a hallmark of HELLP but characteristic feature of severe preeclampsia as well [87]. Hemolysis in preeclampsia may further enhance the platelet activation since breaking red blood cells release ADP [88]. Besides, damaged erythrocytes show an enhanced aggregability as well [89]. This way altered hemostasis with activated platelets markedly contributes to the collapse of microcirculation resulting in tissue hypoperfusion with subsequent end-organ dysfunction in serious preeclampsia.

Platelets actively take part in the defensive action against pathogenic microorganisms. Platelets quickly accumulate and become activated at the site of invasion [90]. They not only release effective agents to damage invading cells but also capable of eliminate them from the circulation. The cationic thrombocidins, releasing from the α granules, are able to depolarize bacterial membranes and inhibit the synthesis of some intracellular molecules [91]. Besides, secondary to stimuli by ADP or stromal cell-derived factor-1 platelets can eliminates bacteria or viruses by internalization (“engulfment”) [92]. The relationship between preeclampsia and infection was posed by the preeclampsia model based on low-dose endotoxin infusion in pregnant rats [93]. Since then several studies found correlation between preeclampsia/HELLP syndrome and different forms of infection:

• Concomitant urogenital infection during pregnancy enhances the incidence of preeclampsia [94];

• Statistical data confirm that preeclampsia incidence increases when patients had *Chlamydia pneumoniae* infection previously [95];

• Periodontitis during pregnancy predisposes for preeclampsia [96];

• Use of antibiotics during pregnancy decreases the incidence of preeclampsia [97];

• In patients with HELLP syndrome a concomitant infection is frequent [98];

It is tempting to assume that the link between preeclampsia and different inflammations may be further enhancing of platelet activation. This presumption seems to be supported by the modest but obvious efficacy of aspirin in the prevention of preeclampsia [99]. Besides, bacterial endotoxin in known to destroy endothelial cells enhancing their dysfunction. Furthermore, platelet adhesion and aggregation could be the consequence of elevated shear stress [100]. High shear stress is ordinarily caused by hypertension, which is a criterion of preeclampsia.

Platelet activation in HELLP syndrome has similar background than it is characteristic for severe preeclampsia; however, there are some differences. The early trophoblast lesion is more pronounced in HELLP. The antiangiogenic sEng levels are significantly higher in HELLP than in preeclampsia. Development of microangiopathy, secondary to increased sEng, sFlt-1, activated TNFα, and von Willebrand factor in HELLP may explain the extreme platelet consumption as consequence of overstimulation [63].

In severe preeclampsia and HELLP syndrome platelets are highly stimulated. Falling the platelet count and elevation of platelet aggregation products precede the manifestation of clinical symptoms by several weeks [75]. This stimulation could be enhanced further by concomitant infection which worsens the condition. Inhibition of platelet aggregation, starting in first trimester, may have a beneficial effect. On the contrary, when the consumption permanently exceeds the production the platelet-associated regulatory system (PARS) collapses and the condition turns to critical state.

## Conclusion - PARS

The above summarized data and the other relevant literature have proved that blood platelets play an essential role in different regulatory processes; among others hemostasis and thrombosis, inflammatory reactions, malignancies, immune responses and in endocrine regulation. Platelets have an armament of soluble molecules, factors, mediators, chemokines, cytokines and neurotransmitters in their granules, and have multiple adhesion molecules and receptors on their surface. Considering that platelets are indispensable in numerous homeostatic endocrine functions, it is reasonable to suppose that a ***platelet-associated regulatory system (PARS)*** may exist; internal or external triggers and/or stimuli may complement and connect regulatory pathways aimed towards target tissues and/or cells. The signal (PAF, or other tissue/cell specific factors) comes from the stimulated (by the e.g., pituitary hormones, bacteria, external factors, etc.) organs or cells, and activates platelets. Platelet activation means their aggregation, sludge formation, furthermore the release of the for-mentioned biologically very powerful factors, which can locally amplify and deepen the tissue specific cell reactions. If this process is impaired or inhibited for any reason, the specifically stimulated organ shows hypofunction. When PARS is upregulated, organ hyperfunction may occur that culminate in severe diseases.

Therefore we suggest that platelets are not only a bystander but rather may be the conductor or active mediator of this complex regulatory system which has several unrevealed mechanisms. In other words platelets are corpuscular messengers, or are more than a member of the family providing hemostasis.

**Figure 1:**
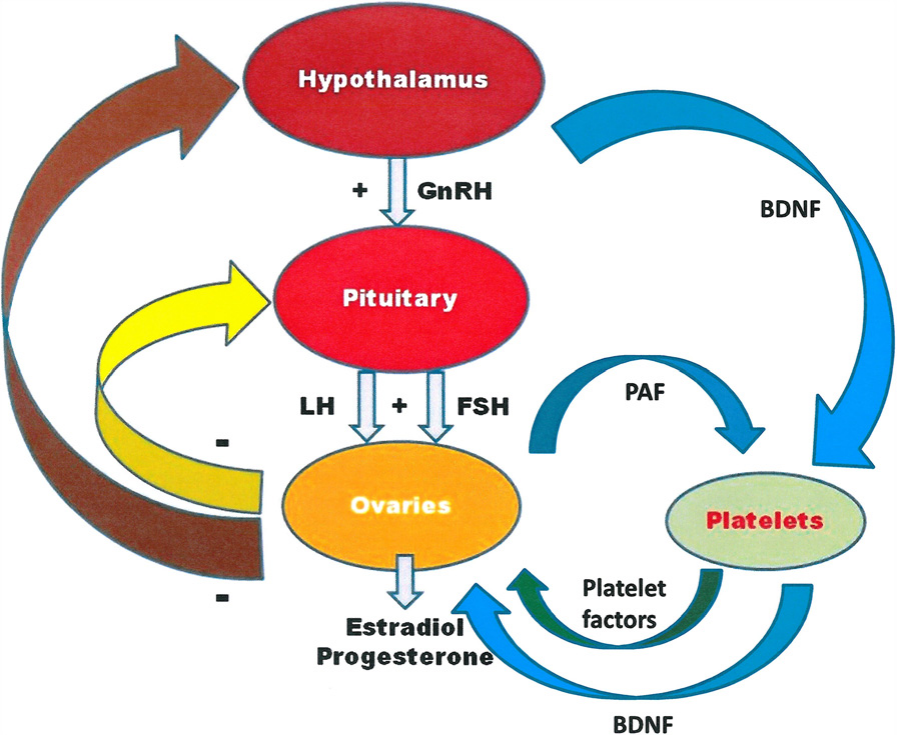
**Hypothetical scheme of platelet-associated regulatory system (PARS).** Note, platelets convey information from the central nervous system to the ovaries via storing and releasing brain-derived factors. GnRH: gonadotropine releasing hormone; LH: luteinizing hormone; FSH: folliculus stimulating hormone; PAF: platelet activating factor; +: stimulation; − : inhibition, BDNF: brain-derived neurotrophic factor.

The possible existence of PARS has been established by detailed analysis of relevant litterature, however further targeted studies should be conducted to confirm the concept delineated in this article (Figure 1).

## Abbreviations

ADMA: Asymmetric dimethyl arginine; ATP: Adenosine triphosphate; ADP: Adenosine diphosphate; BDNF: Brain-derived neutrophic factor; C1qR: C1q‒receptor; CD40L: CD40 ligand; COX: Cyclooxygenase; CRP: C‒reactive protein; DC-SIGN: Dendritic cell-specific intercellular adhesion molecule-3-grabbing non-integrin; DHEA: Dehydroepiandrosterone; EGF: Epidermal growth factor; F11R: F11 receptor; GP Ib-IX: Glycoprotein Ib-IX; GP IIb-IIIa: Glycoprotein IIb-IIIa; HELLP: Low platelet count syndrome; LLP-3: Lipid-phosphatase 3; ICAM-2: Intercellular adhesion molecule 2; IP-10: Human interferon-inducible protein 10; IUGR: Intrauterine growth retardation; MPs: Microparticles; NK: Natural killer cell; NO: Nitric oxide; OHSS: Ovarian hyperstimulation syndrome; PAF: Platelet-activating factor; PARS: Platelet associated regulatory system; PDGF: Platelet-derived growth factor; PF4: Platelet factor 4; PGI2: Prostaglandin I2; PlGF: Placental growth factor; PMPs: Platelet microparticles; PREG: Pregnenolone; PSGL-1: P-selectin glycoprotein ligand-1; sEng: Endoglin; sFlt-1: Fms-like tyrosine kinas-1; TF: Tissue factor; TGFβ: Transforming growth factor beta; TLRs: Toll-like receptors; TNF-α: Tumor necrosis factor α; TXA2: Thromboxane A2; VEGF: Vascular endothelial growth factor; vWF: Von Willebrand factor.

## Competing interests

The authors declare that they have no competing interests.

## Authors’ contributions

All authors read and approved the final manuscript.
